# Application of Machine Learning in Pulmonary Function Assessment Where Are We Now and Where Are We Going?

**DOI:** 10.3389/fphys.2021.678540

**Published:** 2021-06-24

**Authors:** Paresh C. Giri, Anand M. Chowdhury, Armando Bedoya, Hengji Chen, Hyun Suk Lee, Patty Lee, Craig Henriquez, Neil R. MacIntyre, Yuh-Chin T. Huang

**Affiliations:** ^1^Division of Pulmonary and Critical Care Medicine, Loma Linda University Medical Center, Loma Linda, CA, United States; ^2^Division of Pulmonary, Allergy and Critical Care Medicine, Duke University Medical Center, Durham, NC, United States; ^3^Department of Mechanical Engineering and Materials Science, Pratt School of Engineering, Duke University Medical Center, Durham, NC, United States; ^4^Hartford HealthCare, Hartford, CT, United States

**Keywords:** pulmonary function test, flow-volume loop, machine learning, artificial intelligence, spirometry, lung volumes, DLCO

## Abstract

Analysis of pulmonary function tests (PFTs) is an area where machine learning (ML) may benefit clinicians, researchers, and the patients. PFT measures spirometry, lung volumes, and carbon monoxide diffusion capacity of the lung (DLCO). The results are usually interpreted by the clinicians using discrete numeric data according to published guidelines. PFT interpretations by clinicians, however, are known to have inter-rater variability and the inaccuracy can impact patient care. This variability may be caused by unfamiliarity of the guidelines, lack of training, inadequate understanding of lung physiology, or simply mental lapses. A rules-based automated interpretation system can recapitulate expert’s pattern recognition capability and decrease errors. ML can also be used to analyze continuous data or the graphics, including the flow-volume loop, the DLCO and the nitrogen washout curves. These analyses can discover novel physiological biomarkers. In the era of wearables and telehealth, particularly with the COVID-19 pandemic restricting PFTs to be done in the clinical laboratories, ML can also be used to combine mobile spirometry results with an individual’s clinical profile to deliver precision medicine. There are, however, hurdles in the development and commercialization of the ML-assisted PFT interpretation programs, including the need for high quality representative data, the existence of different formats for data acquisition and sharing in PFT software by different vendors, and the need for collaboration amongst clinicians, biomedical engineers, and information technologists. Hurdles notwithstanding, the new developments would represent significant advances that could be the future of PFT, the oldest test still in use in clinical medicine.

## Introduction

The application of machine learning (ML) in medicine is burgeoning and its use in healthcare is potentially transformative (Deo, 2015; Rajkomar et al., 2019). ML is a sub-discipline of computer science in which computers “learn” from large quantities of data in order to find patterns without being explicitly programmed to do so (Sidey-Gibbons and Sidey-Gibbons, 2019). There are two main types of ML—supervised and unsupervised learning. Supervised learning occurs when inputs are chosen with the goal of predicting a known output or target. Typical examples are the development of predictive models that can identify suspicious pulmonary nodules on chest imaging (Uthoff et al., 2019), predict Framingham Risk Score (Chen et al., 2020), and interpret EKGs (Chang et al., 2020). In unsupervised learning, there are no outputs to predict. Instead, the goal is to find naturally occurring patterns or groupings within data. This method has been employed, for example, to identify different phenotypes of sepsis (Seymour et al., 2019). Analysis of pulmonary function test (PFT) is an area where ML, supervised and unsupervised, may benefit the clinicians and the patients (Mlodzinski et al., 2020). The ML methods that may be useful in the analysis of numeric and graphic data of PFTs are shown in the Table 1. To understand how, it is helpful to first examine the current pitfalls in PFT interpretation.

**TABLE 1:** Machine learning methods that may be useful in the analysis of the numeric and graphic data of the pulmonary function tests.

Methods	Description
Random forests	A method of decision tree analysis in which a supervised algorithm works through “bagging” approach to create multiple decision trees with a random subset of the data. These decision trees are then merged to get a more accurate and stable prediction. It is the most common machine learning technique and is best suited for classification and regression tasks.
Neural network	A set of algorithms that uses interconnected layers of computational units (analogous to neurons in the brain) to find relationships in data by iteratively adapting the weights between units. The network typically consists of an input layer that receives the data, several hidden layers, and an output layer. The network can learn using supervised training where an input/output relationship is known or through unsupervised training where no outputs are provided.
Convolutional neural network	A form of neural network, in which the network learns to optimize the filters (or kernels) that slide along input features through automated learning and provides translational responses. It is most applied to analyze visual images.
Fuzzy logic	A means of fuzzy mathematics that is best suited to handle partial truth where the truth value of the variables may be any real number between 0 and 1. The method has the capability of recognizing, interpreting, and utilizing data and information that are vague and imprecise, and outputs the degrees of truth. The reasoning style fuzzy system can be combined with the learning structure of neural networks to become fuzzy-neural systems. The hybrid intelligent system has the strength of incorporating the universal approximation theorem to discover the interpretable IF-THEN fuzzy rules.
Naïve Bayes	A probabilistic classifier based on Bayes’ theorem. It assumes that the value of a particular feature is independent of the value of any other feature. It is a simple technique that only requires a small number of training data to estimate the parameters necessary for classification. The naive Bayes model can be used without accepting Bayesian probability or using any Bayesian methods.
Support vector machine	A supervised machine learning that analyzes data for classification and regression analysis. It can build a model that assigns new examples to one category or the other, making it a non-probabilistic binary linear classifier. It can also perform a non-linear classification using the “kernel trick” mapping the inputs into high-dimensional feature spaces.
k-means clustering	A common unsupervised machine learning method, in which unsupervised algorithms aim to group input vectors into k clusters based on k averages of points (i.e., centroids) without referring to known, or labeled outcomes.
Adaptive Boosting (AdaBoost)	A statistical classification algorithm that is frequently used with other “weaker” machine learning algorithms (e.g., decision tree) to improve their performance. AdaBoost when used with decision trees is often referred to as the best out-of-the-box classifier. The AdaBoost basically improve the relative “hardness” of each learner and converge them to a stronger learner.

### PFT Interpretations Today Use Discrete Data Points and Are Guided by Rules-Based Algorithms

The most common pulmonary function tests (PFTs) are spirometry, lung volume determinations, and diffusing capacity assessments. These tests have two fundamental goals: (1) describe/categorize physiologic abnormalities in a subject and (2) quantify the magnitude of the abnormality. Physiologic abnormalities are conventionally grouped as obstructive ventilatory defects, restrictive ventilatory defects, and gas transfer defects, though multiple defects can be found in a single test. These are currently defined by whether certain discrete measurements are within a pre-determined normal range of values (“rules based” interpretations). The magnitude of the defect is quantified by either reporting a percent predicted of a reference value or else describing the degree of statistical deviation from the mean predicted value (i.e., z-scores). Importantly, PFT interpretation alone cannot diagnose disease states—this can only be accomplished by incorporating PFT results into the overall clinical picture.

Guidelines have been published to assist in interpretating PFTs (Pellegrino et al., 2005). PFT interpretations by clinicians, however, are known to have significant inter-rater variability (Miller et al., 2011; Holt et al., 2019; Topalovic et al., 2019b) and the inconsistency can potentially impact patient care (Enright, 2006; Holt et al., 2019). This variability is due to multiple factors: (1) disagreement among interpreters regarding the choice of reference values and cut-points, (2) actual quality of test performance (and the recognition of this by interpreters), (3) inherent uncertainty about the significance of borderline values, and (4) human errors from lack of training, inadequate understanding of lung physiology, or simply mental lapses.

A rules-based expert computerized interpretation system can help standardize interpretation criteria and address human factors thereby decreasing inter-rater variability and improving the quality and consistency of PFT interpretation. This is not a new concept. Indeed, automated algorithms developed as early as the 1980s sought to reproduce the assessments of an expert physician. For example, a rules-based expert system, PUFF, was developed for local use. It aimed to capture expertise knowledge in PFT interpretation and reduce the tedious work for the clinicians (Aikins et al., 1983). The agreement between PUFF and the physicians was excellent (89–96%), but the continued use of PUFF was hampered by incompatibility when it was modified for other network systems. Another interpretation program was developed using a least mean squares method to rapidly analyze the patient’s PFT data. It was able to select the “best interpretation statements” that were acceptable to a pulmonary physician in 90% of the patients (Krumpe et al., 1982). In one study, the pattern recognition of PFTs by pulmonologists matched the guidelines in about 75% of the cases (Topalovic et al., 2019a). An AI-based software perfectly matched the PFT pattern interpretations (100%) and assigned a correct diagnosis in 82% of all case (Topalovic et al., 2019a). Today, modern PFT systems often offer automated interpretive features based on recommendations such as those proposed by the ATS/ERS in 2005 (Pellegrino et al., 2005), but the features vary significantly and are not validated. Importantly, any rules-based expert computerized interpretation systems should not be considered a replacement for human input because there are still patient and technical factors that may affect the data’s suitability for interpretation by an algorithm.

### Using ML to Go Beyond Simply Automating Interpretation Rules

**FIGURE 1:**
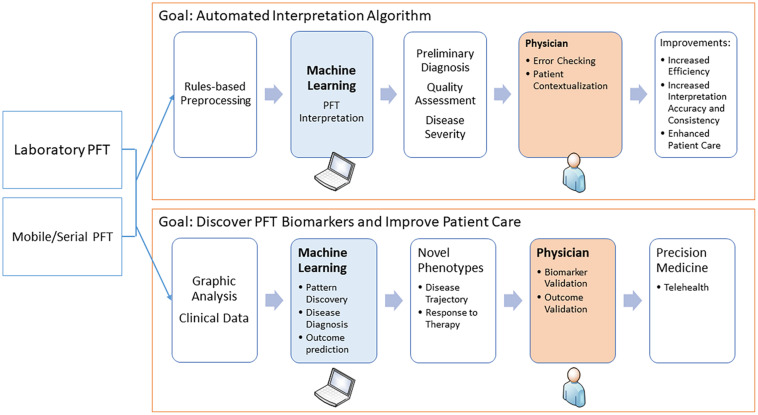
The diagram shows how machine learning may be used in pulmonary function testing. The two main areas are to assist in the interpretation and to discover novel physiological biomarkers.

The potential for using ML in PFT interpretation is expanding in several directions. First, ML is being used to better detect technical deficiencies and poor-quality data to avert algorithm misclassifications and alert the interpreters. Second, attempts are being made to combine PFT data with the clinical picture to better diagnose specific disease states. Third, and perhaps most exciting, ML is being used to analyze continuous data, not just discrete data points, to define new patterns of physiologic dysfunction and links to disease states. Finally, ML can be used to integrate PFT data into the realm of telehealth. These are discussed in detail below and outlined in the Figure 1.

#### Improving Test Quality

Standard PFTs require properly calibrated equipment, standardized testing procedures, and cooperative patients. Current ERS/ATS standards define test quality using checklists filled out by the technologists. Checklists, however, are poor at assessing many of the nuances associated with good patient effort and subtle machine performance characteristics. ML techniques could aid in assessing the quality of the forced expiratory flow pattern, inert gas washout pattern, panting maneuvers, and breath-holds required during standard PFTs. These possibilities have yet to be developed in any practical fashion.

#### Incorporating PFT Patterns With Clinical Data to Formulate Diagnostic Possibilities

Although ML algorithms can be developed to learn to simply recapitulate the multitude of patterns already known to and used by experts, an exciting challenge is using ML to explore existing PFTs in conjunction with the available healthcare data to potentially uncover completely novel associations between PFT patterns and diseases. A simple example available today is a decision tree model that incorporates lung function and clinical variables to improve the accuracy for detecting common lung diseases including COPD, asthma, interstitial lung disease, and neuromuscular disorder, compared to using PFT data alone (Topalovic et al., 2017). This study included 968 new patients seen in a pulmonary practice. The pulmonary diagnoses of these patients were labeled based on the combination of PFT results and the physician’s assessment. It was found the ATS/ERS algorithm resulted in a correct diagnostic label in 38% of the patients. COPD had the highest positive predictive value (74%), whereas all other diseases were poorly identified. The decision tree algorithm improved the overall accuracy by ~ 2-fold (68%) with an improved positive predictive value for COPD (83%), asthma (66%), interstitial lung disease (52%), and neuromuscular disorder (100%). Another study that used a neuro-fuzzy system incorporating spirometric parameters (FEV_1_, FVC, and FEV_1_/FVC) and clinical symptoms was able to classify asthma and COPD with >99% accuracy (Badnjevic et al., 2015).

#### Analyzing Continuous Data

ML, supervised and unsupervised, can also be used to go beyond identifying patterns described by discrete data points and analyze continuous data or graphics. A prime example would be an analysis of the entire expiratory flow-volume loop (FVL). The traditional approach to assess the forced expiratory portion of the FVL uses a small number of discrete values, such as FEV_1_, FVC, FEF_2__5__–__7__5__%_, and the FEV_1_/FVC ratio. FEV_1_ is the most reproducible spirometric parameter reflecting bronchial caliber, yet it is unable to quantify ventilation inhomogeneity (Ross et al., 1992). FEF_2__5__–__7__5__%_ is quite variable and is not reliable for the diagnosis of small airway disease (the so-called “quiet zone”) (Quanjer et al., 2014; Malerba et al., 2016). Since the expiratory flow signals in a FVL reflect the sequential emptying properties of multiple lung units having different time constants as the lung volume decreases, analysis of the entire curve would be a better method to explore regional lung properties such as small airway function and mechanical (i.e., compliance and resistance) heterogeneity (Topalovic et al., 2014).

Numerous approaches to analyzing the FVL have been reported. An artificial neural network model that combined traditional spirometric measurements and area under the expiratory flow-volume curve differentiated well between normal, obstruction, restriction, and mixed impairments (Ioachimescu and Stoller, 2020). The square root of the area under the expiratory flow-volume curve plus FEV_1_, FVC, and FEV_1_/FVC z-scores could categorize ventilatory impairments with very low rates of misclassification (<9%) compared with standard classifications based on FVC, FEV_1_/FVC, and TLC (Ioachimescu and Stoller, 2020). A fully convolutional neural network of flow-volume curves with CT scan as the output was more accurate in discriminating predominant emphysema/airway phenotypes in COPD (area under the curve [AUC] = 0.80) compared with traditional spirometry parameters (FEV_1_ [area under the curve, or AUC = 0.71] and FEV_1_/FVC [AUC = 0.70]) and random forest classifier (AUC = 0.78). The neural network was also better in discriminating predominant emphysema/small airway phenotypes (AUC 0.91) compared with FEV_1_/FVC (AUC 0.80), FEV_1_% predicted (AUC 0.83), and had similar accuracy to random forest classifier (AUC 0.90) (Bodduluri et al., 2020). Geometric analysis of the expiratory FVL demonstrated the concavity of the forced expiratory curve quantified by a slope-ratio was more prominent in asthmatic patients (1.35 ± 0.03) than normal subjects (0.90 ± 0.11) (Dominelli et al., 2016). The shape factors at 50 and 75% FVC and the slope ratio at 75% FVC improved after inhaled corticosteroid treatment (Kraan et al., 1989). The concavity of the expiratory limb of the spontaneous FVL measured by a rectangular area ratio obtained during exercise was inversely correlated with dynamic hyperinflation and exercise limitation in patients with COPD (Varga et al., 2016). The parameters in a second-order transfer function model of the flow dynamics during forced expiration (the two poles and the steady state gain) identified patients with COPD defined by the existing guidelines with high accuracy (88.2%) and may help in cases where FEV_1_/FVC ratio-based diagnosis is uncertain (Topalovic et al., 2014). The multiscale computational modeling, which is the use of computers to simulate and study complex systems using mathematics, physics, and computer science, can be used to analyze expiratory flow in FVL and help evaluate obstructive lung diseases (Burrowes et al., 2014). Incorporating ML and computational modeling into the analysis of the entire FVL is a fruitful avenue for future research.

Evaluation of regional D_LCO_ may also be an application in which ML can be useful. Traditional approaches to measuring D_LCO_, including single-breath, steady-state, and rebreathing methods, treat the lung as a single, well-mixed compartment and produce a single value of D_LCO_ that is taken to represent an average D_LCO_ at a given lung volume. Currently the single breath D_LCO_ measures are calculated by the changes in the concentration of CO relative to an inert gas in one alveolar sample in the expired gas (Graham et al., 2017). In fact, the entire exhalation curves of CO and inert gas after the breath hold can be modeled and analyzed with the help of ML. This could shed light on the non-uniform distribution of D_LCO_ due to the heterogeneity of blood flow and ventilation distribution that is best observed in slow exhalation, when D_LCO_ is found to decrease non-linearly from high to low lung volumes (Stokes et al., 1981; Huang et al., 2002). Like the FVL, ML analysis of the entire exhalation curve could improve insight into regional changes in D_LCO_ over single summary measurements, and provide earlier diagnosis of lung disease, such as emphysema and pulmonary vascular disease. Similarly, ML can also be used to analyze the nitrogen washout curve and data from forced oscillation technique. Such analyses potentially allow for the discovery of novel clinical and physiological phenotypes related to emptying properties of different lung regions (or ventilation heterogeneity) in obstructive lung diseases independent of traditional spirometry indices (Mikamo et al., 2013; Timmins et al., 2014).

#### Intergrating PFT Data Into Telehealth Applications

In the era of wearables and telehealth, remote respiratory monitoring for chronic respiratory patients has been proposed to reduce hospitalizations, improve self-care, and enhance health-related quality of life. The need for telemonitoring has become more essential with the COVID-19 pandemic restricting in-person pulmonary function testing (Kouri et al., 2020). Handheld spirometers are among the respiratory devices that are suitable for telemonitoring application. The spirometric data can be transmitted via a gateway (e.g., a smartphone) to a cloud repository site. ML may have increased application opportunities in screening and interpreting these mobile pulmonary function data. A smartphone game-based pulmonary function assessment has demonstrated good correlation with those measured by a spirometer in 34 stroke patients with intraclass correlation coefficients for FVC and FEV_1_ of > 0.90 (Joo et al., 2018). FEV_1_ and FVC measured by a smartphone-connected spirometer agreed with those measured by a conventional spirometer in pediatric patients with cystic fibrosis and asthma (Pearson correlation coefficients > 0.9 for FEV_1_ and FVC), but only less than half of the tests were acceptable and reproducible according to the ATS/ERS criteria (Kruizinga et al., 2020). An automated mobile expert diagnostic telehealth system that consists of a spirometer, mobile application, and expert diagnostic system was able to diagnose asthma and COPD with high accuracy (>90%) in 780 patients at remote primary healthcare institutions and hospitals (Gurbeta et al., 2018). An ML algorithm combined with sociodemographic, clinical, and physiological telemonitoring data was better in predicting acute exacerbations of COPD than the two traditional symptoms-counting algorithms (AUC of 0.74 with the ML algorithm vs. 0.60 and 0.58 for the traditional algorithms) (Orchard et al., 2018). ML may be used to extract usable spirometry data from the mobile programs in the wearable devices. Finally, precision medicine can be delivered by tailoring analyses to an individual’s clinical profile and learning from the experience of a patient (Franssen et al., 2019).

## Hurdles for Development of ML-Based PFT Programs

Despite the exciting potential for the use of ML in PFT, there are hurdles in the development and commercialization of the ML-assisted PFT interpretation programs. These include: (1) the need for high quality representative data, (2) the existence of inherent biases in historical data, (3) the need for development and constant update of validated endpoints on which to train ML models, (4) the existence of different formats for data acquisition and sharing in PFT software by different vendors, and (5) the need for collaboration amongst clinicians, biomedical engineers, and information technologists to acquire large data sets to develop and validate such algorithms. Our medical system is reluctant to entrust a machine with a task that a human can do, particularly due to the “black-box” nature of fully automated systems. In recognition of these hurdles and the need to regulate the development, production and monitoring of ML models in medical devices, the US Food and Drug Administration (FDA) in April 2019 published a discussion paper that proposed a framework for AI/ML-based Software as a Medical Device (SaMD) (USFDA, 2019). The FDA purports to risk stratify SaMDs based on the intended use of SaMD-derived information for healthcare decision making and the risk profile of the individual patient. Based on this stratification, ML-based PFT software could be used for “treating and diagnosing,” “driving clinical management” as well as “informing clinical management” in all tiers of risk, “critical,” “serious,” and “non-serious” (USFDA, 2019). The American Society of Mechanical Engineers has also introduced standards for the ML-based PFT software as an SaMD (V&V40) and machine learning (V&V70) (ASME, 2021). There are working groups on patient-specific models, computational modeling of medical devices, and machine learning under V&V40. This standard can be used by the practitioner as a framework to assess the device/software using sound engineering judgment. Such a nationally accepted framework would be key to addressing quality, liability, privacy, reimbursement, and regulatory issues of AI/ML-based software and may serve to provide an impetus for amalgamations of an integrated man-and-machine approach into daily medical practice.

Hurdles notwithstanding, there is significant potential for the use of ML in pulmonary function assessment. Future research should focus on how ML may improve, simplify, enhance, and expedite PFT interpretation, and integrate PFT parameters with imaging and clinical information to discover novel physiological markers that can enhance the diagnostic sensitivity and specificity of pulmonary diseases. All these developments would represent significant advances that could be the future of PFTs, the oldest test still in use in clinical medicine (MacIntyre, 2012).

## Data Availability Statement

The original contributions presented in the study are included in the article/supplementary material, further inquiries can be directed to the corresponding author/s.

## Author Contributions

PG, AC, and Y-CH: develop the concept and write the draft and edit the manuscript. AC, AB, CH, HL, PL, and HC: provide discussion on the machine learning and edit the manuscript. PG, NM, and Y-CT: provide discussion on the pulmonary function test and edit the manuscript. All authors contributed to the article and approved the submitted version.

## Conflict of Interest

The authors declare that the research was conducted in the absence of any commercial or financial relationships that could be construed as a potential conflict of interest.
